# A new modified Duckett urethroplasty for repair of proximal hypospadias with severe chordee: outcomes of 133 patients

**DOI:** 10.1186/s12894-022-00993-x

**Published:** 2022-03-28

**Authors:** Chong Wang, Zhi-Cheng Zhang, De-Ying Zhang, Yi Hua, Feng Liu, Da-Wei He, Guang-Hui Wei, Xing Liu

**Affiliations:** Department of Urology, Children’s Hospital of Chongqing Medical University, Ministry of Education Key Laboratory of Child Development and Disorders, National Clinical Research Center for Child Health and Disorders, China International Science and Technology Cooperation Base of Child Development and Critical Disorders, Chongqing Key Laboratory of Children Urogenital Development and Tissue Engineering, 136 Zhongshan Road, Chongqing, 400010 China

**Keywords:** Proximal hypospadias, Penile chordee, Duckett urethroplasty, Urethral plate, Flap

## Abstract

**Background:**

Despite the continuous development and evolution of surgical methods and techniques, proximal hypospadias remains one of the most challenging issues for pediatric urologists. This study aims to evaluate the indications and postoperative complications of our new modified Duckett urethroplasty.

**Methods:**

A total of 133 patients with proximal hypospadias who underwent repair of the modified Duckett urethroplasty from February 2016 to February 2021 were reviewed. The median age of patients was 3 years (range 1–16). All patients had severe chordee. One senior experienced pediatric urologist performed all the surgeries. Catheter was removed 14 days after the surgery.

**Results:**

The location of the urethral meatus was proximal penile in 26 patients (19.5%), penoscrotal in 60 (45.1%), scrotal in 31 (23.3%), and perineal in 16 (12.0%). The mean length of the urethral defect was 4.5 cm (range 2.5–10). The median duration of follow-up was 46 months (range 8–67). Complications occurred in 31 patients (23.3%), including urethra-cutaneous fistula in 22 (16.5%), urethral stenosis in 7 (5.3%), and urethral diverticulum in 2 (1.5%). No recurrent chordee were found in all cases. All patients who developed complications were treated successfully at our hospital.

**Conclusions:**

Our modified Duckett urethroplasty showed functionally and cosmetically favorable outcomes, with a lower incidence of postoperative complications. To the best of our knowledge, the novel Duckett technique is a feasible and suitable option for patients who suffer from proximal hypospadias with severe chordee and dysplasia of the urethral plate.

## Background

Hypospadias is one of the most common congenital urogenital abnormalities. Although various techniques like Onlay, Duckett and Koyanagi technique were created to correct hypospadias, it is very important to choose the most reliable surgical method, especially for severe hypospadias. As the most challenging type of hypospadias, arguing about appropriate surgical procedure of proximal hypospadias with severe chordee never stops. Compared with distal and mid-penile hypospadias, the incidence of complications and re-operation rates of proximal hypospadias were obviously higher [1, 2]. Transverse Preputial Island Flap (TPIF) urethroplasty, also known as Duckett technique, is wildly used for repair of proximal hypospadias. However, the procedure is difficult and the complication rate is high even in skilled hands [3–6]. Therefore, how to simplify the surgical procedure and reduce the incidence of complications remained a challenge for pediatric urologists.

Recently, we have obtained experiences in performing our modified Duckett urethroplasty. We have modified the surgical procedures regarding tubularization of flap, urethral anastomosis, and extension of the indwelling catheter time to improve the surgical fluency and reduce complications. The surgeon who performed all the surgeries in this study practiced the new Duckett approach since 2014. In the present study, we report the postoperative outcomes of modified Duckett technique for the treatment of proximal hypospadias with severe chordee.

## Methods

### Patients

Retrospectively, we reviewed 133 patients with hypospadias who underwent the modified Duckett urethroplasty between February 2016 and February 2021 in our department. Patients with the location of urethral meatus which were proximal penile, penoscrotal, scrotal and perineal were included in this study. All of the 133 patients had severe penile chordee which is defined by penile curvature more than 30° after degloving during the surgery using an artificial erection test. All surgeries were performed by an experienced pediatric urologist who has a stable learning curve of 60 cases of Duckett urethroplasty [7]. We excluded re-operative hypospadias, lack of operation details, incomplete follow-up cases (less than 6 months of follow-up). Preoperatively, skin preparation was performed with 5% povidone-iodine for 3 days. Ethical approval was given by the medical ethics committee of Children’s Hospital of Chongqing Medical University.

### Surgical procedures

A longitudinal incision on the dysplastic distal urethra was performed from the urethral orifice to the bifurcation of the corpus cavernosum. A U-shaped incision around the new urethral orifice was made along the outer edge of the urethral plate, and then a circular cut was made 0.5 cm below the coronal sulcus. Complete degloving of penile skin was carried out, while the dense fascia was fully dissected and loosened until the urethral cavernous muscle was revealed.

In order to evaluate the degree of penile curvature and shortening of the urethra, an artificial erection was induced. In all cases, the curvature was severe enough to perform a transection of urethral plate 0.5 cm below the coronal sulcus. The urethral plate was posteriorly dissected close to the corpus cavernosum albuginea. At the same time, all the fibrous bands longitudinally distributed in the superficial layer of the tunica albuginea were removed. An artificial erection was induced again. If there remained curvature of the corpus cavernosum, a dorsal tunica albuginea plication was performed.

**Fig. 1:**
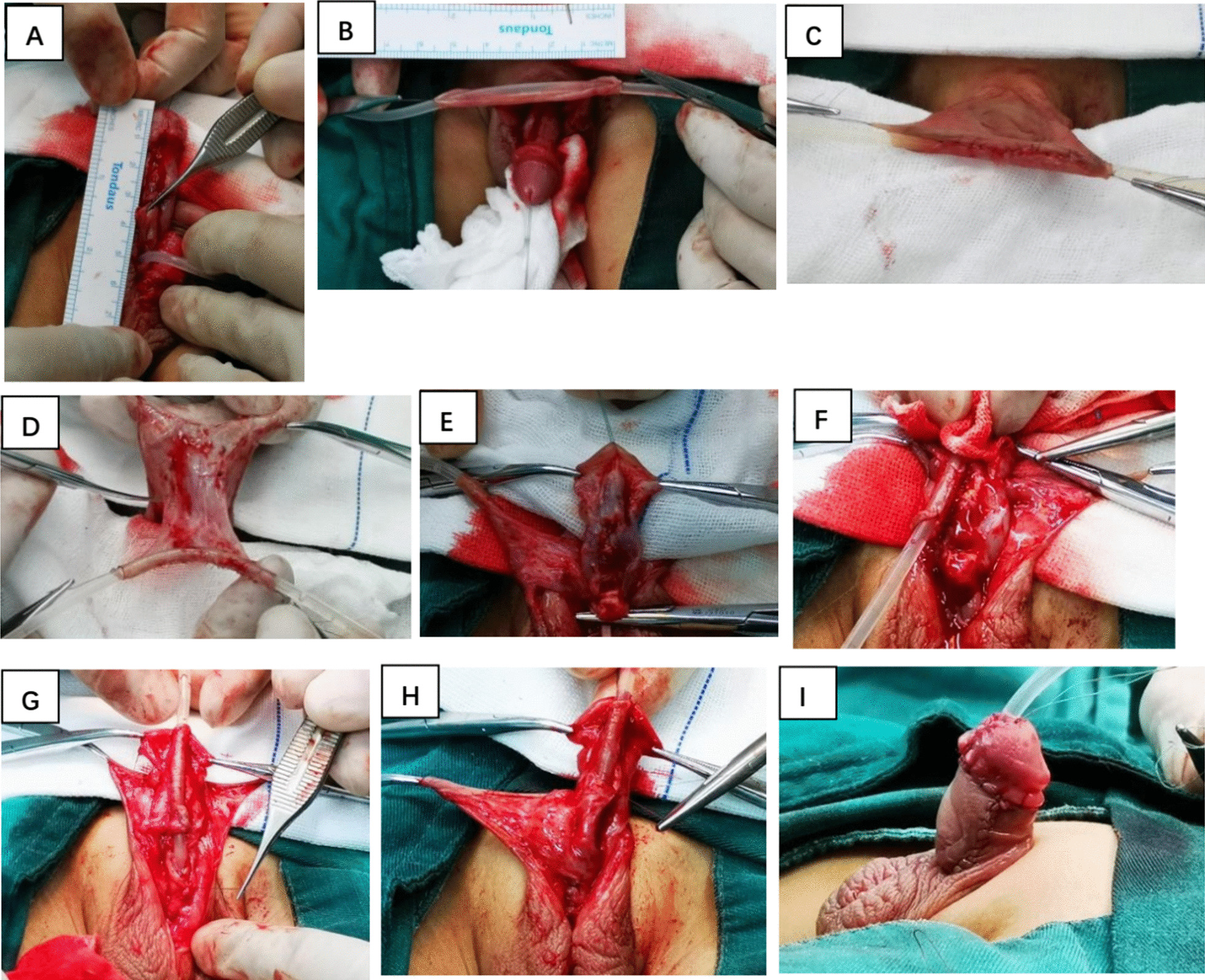
**a** Measure the urethral defect. **b** The two ends of the preputial inner plate were sutured around the catheter. **c** Obtain a complete tubularized tube. **d** The fascial pedicle was separated carefully to obtain a transverse tubularized flap tube. **e** The bilateral glanular wings were dissected along the superficial layer of the albuginea. **f** Refixation of the urethral plate. **g** Anastomosis of tubularized flap tube and original urethral meatus. **h** The fascia pedicle of flap was tacked in place covering the anastomosis and part of the neourethra. **i** Postoperative appearance after the surgery

Next, the length from the tip of the glans to the proximal urethral meatus was measured by ruler, which is the length of the urethral defect (Fig. 1a). We used 8- or 10-Fr catheter as a urethral stent. Taking the length of the urethral defect as a reference, the two ends of the preputial inner plate were sutured around the catheter with 6–0 absorbable suture as a traction (Fig. 1b). The protruding curled inner plate of the prepuce was trimmed to make the edges neat. Interrupted sutures were made on the inner plate of prepuce with a stitch distance of about 2.5 mm. After the tubularization was finished, the epidermal tissue was cut to obtain a complete tubularized tube (Fig. 1c). The fascial pedicle was separated carefully to obtain a transverse tubularized flap tube, while paying attention to protecting the main blood vessels of the skin (Fig. 1d). Afterwards, the tube was transposed on the ventral side of penile body, while the fascial pedicle was tension-free.

Subsequently, the glans was incised in the middle and then the bilateral glanular wings were dissected along the superficial layer of the albuginea (Fig. 1e). The edges and excess tissues were trimmed to fully expand the glanular wings. The neourethra was placed toward the corpus cavernosum. The distal end of the neourethra was sutured to the glans with one stitch, while the proximal end was sutured to the mucosa of the dorsal side the native urethral meatus with 2 stitches after penetrating the albuginea. Refixation of urethral plate was performed while the dorsal side of the anastomosis was formed (Fig. 1f). If the wound of the proximal urethra was long, interrupted sutures were made to tubularize a tube. Sufficient bevel incision was important to be reserved, so as to be aligned and anastomosed with the proximal bevel incision of neourethra by interrupted sutures (Fig. 1g). If the tension of the proximal tubularized tube was too large, a full-thickness longitudinal section on the dorsal side of the urethral mucosa was performed, following the Snodgrass approach.

The fascia pedicle of flap was then tacked in place covering the anastomosis and part of the neourethra, which is beneficial to the survival of the flap and could increase the thickness of the tissue coverage (Fig. 1h). Formation of the glans and the external urethra meatus was performed. The outer plate and the skin on the dorsal side of the prepuce was incised to the level of the coronary sulcus, while care should be taken to avoid damage to the main blood vessels. The skin was turned to the ventral side to suture and wrap the body of the penis. If the patient has a penile-scrotal transposition, correction was not made by force, so as to avoid excessive damage to the blood supply of the skin. Scrotum split was eliminated layer by layer (Fig. 1i). Prophylactic antibiotics were administered for 3 days after the surgery. The penis was covered with dressing for 6 days. Catheter was reserved for 14 days postoperatively.

### Follow-up

The follow-up was organized by the same surgeon who performed all the surgeries, including outpatient follow-ups and telephone interviews. The surgeon assessed the appearance and function of the penis postoperatively. The assessment of appearance included whether there is fistula and recurrent penile chordee, and the assessment of function included that asking the patient or his parents whether the patient had dysuria, weak stream of urine, trickle micturition and penile pain during erection, etc.

## Results

From February 2016 to February 2021, a total of 133 patients were included in our study who were treated by modified Duckett urethroplasty. The median age of patients at the operation was 3 years (range 1–16). After the chordee was thoroughly straightened, the mean length of the urethral defect was 4.5 cm (range 2.5–10). The location of the urethral meatus was proximal penile in 26 patients (19.5%), penoscrotal in 60 (45.1%), scrotal in 31 (23.3%), and perineal in 16 (12.0%). Transection of the urethral plate were performed in all cases because of severe chordee and dysplastic urethral plate. The mean operative time was 91.21 min (range 60–135 min). The median duration of follow-up was 46 months (range 8–67), including outpatient follow-ups and telephone interviews. The basic characteristics and operative details of all patients were summarized in Table 1.

**Table 1:** The basic characteristics and operative details of 133 patients

Characteristic	Summary
Age at surgery (year), median	3.0 (range 1–16)
Urethral defect (cm), mean	4.5 (range 2.5–10)
Type of hypospadias, n (%)	
Proximal penile	26 (19.5%)
Penoscrotal	60 (45.1%)
Scrotal	31 (23.3%)
Perineal	16 (12.0%)
Follow-up duration (month), median	46 (range 8–67)
Operative time(minutes), mean	91.21(range 60–135)
Second follow-up duration (month), median	37 (range 7–60)

**Fig. 2:**
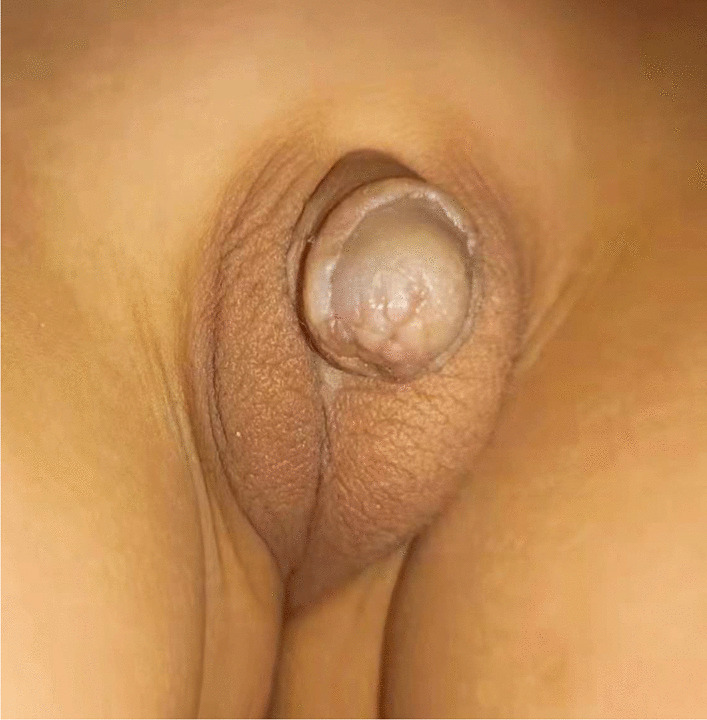
Postoperative picture taken 3 years after our new Duckett urethroplasty

The present study showed that 102 patients (76.7%) were successfully treated after single stage repair (Fig. 2). A total of 31 patients (23.3%) developed complications, of whom 22 (16.5%) had urethra-cutaneous fistula, 7 (5.3%) had urethral stenosis, and 2 (1.5%) had urethral diverticulum (Table 2). No recurrent chordee were found in all cases. In all, further surgical intervention was needed in 30 patients who had postoperative complications. One of the seven patients with urethral strictures were treated by urethral dilatations, while the surgery of urethral reconstruction was performed in the remaining six patients. Fistula were repaired in 22 patients 6 months after the initial operation. Urethral diverticulectomy were performed in 2 patients. All patients suffering from the postoperative complications received medical treatments at our hospital. Follow-ups were also performed on all of 31 patients who had complications. The median duration of second follow-up was 37 months (range 7–60). After surgical and non-surgical interventions, all complications were resolved.

**Table 2:** Complications and the location of the meatus

Complications	Fistula, n (%)	Urethral stricture, n (%)	Urethral diverticulum, n (%)
*Location of meatus*			
Proximal penile	5 (3.8%)	0 (0)	0 (0)
Penoscrotal	13 (9.8%)	4 (3.0%)	1 (0.8%)
Scrotal	4 (3.0%)	2 (1.5%)	1 (0.8%)
Perineal	0 (0)	1 (0.8%)	0 (0)
Total	22 (16.5%)	7 (5.3%)	2 (1.5%)

## Discussion

Generally, the choice of repair technique seems to be largely influenced by the preference of the surgeon and the subjective assessment of the characteristics of hypospadias. Onlay, tubularized incised plate (TIP) and Duckett urethroplasty are trustworthy for their reproductivity and feasibility, which appear to have less complication rate comparing to other procedures [6, 8, 9]. Since Duckett first described the transverse preputial island flap urethroplasty (TPIF), the surgical procedure for repairing hypospadias has been widely used [10]. However, the disadvantage of the operation is that the learning curve required to grasp this technique is steep, as well as intractable complications [11–14].

In terms of improving surgical fluency, we directly tubularized the prepuce inner plate with the urinary tube as a support before harvesting the pedicle flap. Therefore, the operating step is easier. Meanwhile, estimating the width of the flap could be more intuitive, so as to enable a more appropriate diameter of tubularized flap.

As reported in other literature, urethra-cutaneous fistula and urethral stenosis are the most common complications of TPIF urethroplasty. Respectively, the rate of fistula and stenosis can run up to 66% and 44% [6, 15, 16], although the overall complication rate could be as low as 14.6% in proficient hands [17]. In the past years, many authors made well efforts to reduce the developments of complications by modifying the Duckett procedure [8, 9, 18].

Unlike Onlay and TIP repair, one of the defining features of Duckett procedure is the need to transect the urethral plate. Urethral plate (UP) is considered as an ideal material for neourethra, due to the fact that it consists of collagen I and III, nerve tissue, and rich blood vessels [19]. Procedures which reserve UP like Onlay repair could give the neourethra a dual blood supply from both UP and the vascular pedicle, lowering the possibility of ischemia of the flap. Therefore, the rate of fistula and urethral strictures can be reduced. However, preserving UP is not an assurance for decreasing complications. In the findings of Snodgrass and Braga, the overall rate of complications for repair of proximal hypospadias with UP preserved was 35–37% [20, 21]. On the other hand, Onlay urethroplasty is not preferable in cases with severe penile chordee, while the Duckett urethroplasty does. For patients who have severe penile curvature and dysplastic urethral plate, transection of the urethral plate for fully correcting the curvature is essential and inevitable. For this reason, in our study, urethral plate of all the included patients were transected. However, given that the benefit of abundant blood vessels, we preserve the glans part of UP, making the transection at 0.5 cm below the coronal sulcus for the purpose of increasing the blood supply. Afterwards, a re-fixation of UP was performed.

On the other hand, we modified our steps by using interrupted suturing technique instead of continuous suture when we perform the tubularization of preputial flap, in order to get a lower complication rate. From our perspective, compared with continuous suture, the interrupted suture can minimize the curling of the tubularized flap. Accordingly, the incidence of flap ischemia and necrosis could be reduced. Samir et al. conducted a prospective trial to compare the outcome of interrupted suture and continuous suture in hypospadias repair using TIP technique. Their result showed that the interrupted sutures groups was associated with a lower complication rate [22].

Apart from the procedures noted above, we improved the technique while making the anastomosis between the tubularized flap and original urethral meatus. Owing to the intersection of different tissues, the anastomosis between neourethra and native urethral meatus is with a tendency of stenosis [23]. Bevel incision was sufficient and large enough, and then we make the anastomosis by interrupted sutures, in order to release the tension. If the tension of the proximal tubularized tube was too high, a full-thickness longitudinal section on the dorsal side of the urethral mucosa was performed. In addition, the fascia pedicle of flap was then tacked in place covering the anastomosis and part of the neourethra, which is beneficial to the survival of the flap and could increase the thickness of the tissue coverage.

In addition, we expect that extending the indwelling catheter time can reduce the incidence of urethral stricture. All the urinary catheters of patients in our current study were kept for 14 days before being removed. In this way, we believe it can decrease occurrence of urine extravasation, reduce postoperative inflammation and scar hyperplasia. At the same time, it provides a supporting effect, and prevents the neourethra from being angled and twisted during the fusion process of the newly formed urethra and the surrounding tissues. Consequently, the occurrence of urethral stricture could be reduced.

Fortunately, in this study, the rate of fistula and urethral stricture is 16.5% and 5.3% respectively. The number is significantly lower than the reported rate in a 20-year systematic review [6], where the Duckett technique demonstrated a fistula rate of 22.4% and a urethral stricture of 12.5%.

Moreover, in terms of recurrent curvature, fortunately, we got a favorable result that no patient was found recurrent penile chordee. Nevertheless, we shall never neglect the importance of long-term follow-up. Vandersteen reported 22 cases of late onset recurrent chordee after hypospadias repair [24]. All the onset of recurrence developed during puberty with the median age at 16 years, which indicates the necessity of focusing on late onset complications. Hence, longer and continuous follow-up is required necessarily, 52.9% of patients had at least one problem that required long-term follow-up [25]. Secondary surgery rates are underreported if follow-up is limited to less than 6 years [26].

Furthermore, whether choosing single-stage or two-stage urethroplasty for the treatment of proximal hypospadias with severe chordee remains controversial [27]. The two-stage procedure is considered as a method to reduce the difficulty of surgery, in terms of increasing the available prepuce, tissue vascularity and providing a healthier urethral bed for the second stage surgery [28]. Reportedly, it has relatively low risk of complications [6, 27, 29, 30]. However, compared with two-stage urethroplasty, most of the patients can avoid re-operation who undergo a single-stage urethroplasty. In our study, the overall complication rate is 23.3%, thus 76.7% of the patients have benefits over staged procedure, such as lower risk of anesthesia, less cost of treatment, and decreased psychological influence of surgery [31]. Additionally, 22 of 31 patients (71.0%) who had fistula can be treated by a minor surgical repair.

Since this is a retrospective study, a selection bias of surgical methods is inevitable. A prospective study is necessary to be carried out, so that the outcomes ought to be undoubtedly more convincing. Meanwhile, the limitations of this study also include the relatively insufficient follow-up duration, that we are not able to find out the long-term complications like recurrent chordee and sexual intercourse disorders. Moreover, data of uroflowmetry for evaluation of urethral function were not collected in this study. In addition, a comparison group of other repair techniques is essential to help compare and confirm the benefits of our modified technique in the future.

In brief, based on the traditional Duckett technique, we have modified the surgical procedures regarding tubularization of flap, urethral anastomosis, and extension of the indwelling catheter time to improve the surgical fluency and reduce complications. All of the operations were performed by the same experienced doctor who has a stable learning curve of Duckett urethroplasty. In this way, the bias caused by different skill level of different surgeons can be avoided.

## Conclusions

Our modified Duckett urethroplasty showed both functionally and cosmetically favorable outcomes, with much lower incidence of postoperative complications. The novel technique is a suitable option for patients who suffer from proximal hypospadias with severe chordee and dysplasia of the urethral plate.

## Data Availability

All data generated or analyzed during this study are included in this published article. The datasets used and/or analyzed during the current study are available from the corresponding author on reasonable request.

## Abbreviations

UP: Urethral plate
TPIF: Transverse preputial island flap
TIP: Tubularized incised plate
